# Outcomes of a Community Engagement and Information Gathering Program to Support Telephone-Based COVID-19 Contact Tracing: Descriptive Analysis

**DOI:** 10.2196/40977

**Published:** 2022-11-15

**Authors:** Chi-Chi N Udeagu, Masha Pitiranggon, Kavita Misra, Jamie Huang, Thomas Terilli, Yasmin Ramos, Martha Alexander, Christine Kim, David Lee, Kathleen Blaney, Chris Keeley, Theodore Long, Neil M Vora

**Affiliations:** 1 New York City Test & Trace Corps New York City Department of Health & Mental Hygiene Queens, NY United States; 2 New York City Test & Trace Corps New York City Health + Hospitals New York City, NY United States

**Keywords:** COVID-19, contact tracing, home visits, community health workers, health equity

## Abstract

**Background:**

Contact tracing is an important public health tool for curbing the spread of infectious diseases. Effective and efficient contact tracing involves the rapid identification of individuals with infection and their exposed contacts and ensuring their isolation or quarantine, respectively. Manual contact tracing via telephone call and digital proximity app technology have been key strategies in mitigating the spread of COVID-19. However, many people are not reached for COVID-19 contact tracing due to missing telephone numbers or nonresponse to telephone calls. The New York City COVID-19 Trace program augmented the efforts of telephone-based contact tracers with information gatherers (IGs) to search and obtain telephone numbers or residential addresses, and community engagement specialists (CESs) made home visits to individuals that were not contacted via telephone calls.

**Objective:**

The aim of this study was to assess the contribution of information gathering and home visits to the yields of COVID-19 contact tracing in New York City.

**Methods:**

IGs looked for phone numbers or addresses when records were missing phone numbers to locate case-patients or contacts. CESs made home visits to case-patients and contacts with no phone numbers or those who were not reached by telephone-based tracers. Contact tracing management software was used to triage and queue assignments for the telephone-based tracers, IGs, and CESs. We measured the outcomes of contact tracing–related tasks performed by the IGs and CESs from July 2020 to June 2021.

**Results:**

Of 659,484 cases and 861,566 contact records in the Trace system, 28% (185,485) of cases and 35% (303,550) of contacts were referred to IGs. IGs obtained new phone numbers for 33% (61,804) of case-patients and 11% (31,951) of contacts; 50% (31,019) of the case-patients and 46% (14,604) of the contacts with new phone numbers completed interviews; 25% (167,815) of case-patients and 8% (72,437) of contacts were referred to CESs. CESs attempted 80% (132,781) of case and 69% (49,846) of contact investigations, of which 47% (62,733) and 50% (25,015) respectively, completed interviews. An additional 12,192 contacts were identified following IG investigations and 13,507 following CES interventions.

**Conclusions:**

Gathering new or missing locating information and making home visits increased the number of case-patients and contacts interviewed for contact tracing and resulted in additional contacts. When possible, contact tracing programs should add information gathering and home visiting strategies to increase COVID-19 contact tracing coverage and yields as well as promote equity in the delivery of this public health intervention.

## Introduction

Worldwide, the emergence of COVID-19 as a public health crisis prompted a range of measures to curb the spread of SARS-CoV-2, the virus that causes COVID-19. The mitigation measures included nonpharmaceutical interventions, such as handwashing, stay-at-home order, self-masking, social distancing, and limits on the type and number of people at social gatherings [1-4]. In addition, public health jurisdictions applied contact tracing strategies to identify and notify exposed contacts of people with COVID-19 to stem ongoing disease transmission [5-10].

Contact tracing is a resource-intensive, multistep process [7]. The core feature of an efficient and effective contact tracing program is timely case identification and investigation to elicit exposed contacts and ensure self-isolating of case-patients as well as the notification and quarantining of their contacts [11,12]. Studies have found that early identification of cases through testing and contact tracing and quarantining of the exposed contacts can result in about 80% reduction in the transmission SARS-CoV-2, including transmissions by presymptomatic or asymptomatic individuals with COVID-19 [13-15].

Nonetheless, the high burden of COVID-19 cases presented overwhelming challenges to contact tracing programs in reaching every case-patient or contact and conducting timely manual contact tracing via telephone calls [16-19]. Therefore, many public health jurisdictions added digital contact tracing, which involves the use of smartphones to optimize the breadth of tracing and minimize delays in contact notifications [8-10]. Digital proximity contact tracing aims to rapidly identify people who may have been in contact with individuals subsequently diagnosed with COVID-19 for a certain amount of time, using electronic techniques including Bluetooth, Global Positioning System, or Wi-Fi.

Manual telephone calls and digital contact tracing rely on the ownership and use of smartphones, electronic tracking systems, and accurate telephone numbers. People with COVID-19 or their contacts may lack access to telephone or mobile technology or the skill and ability to operate them [20-22]. Furthermore, people with COVID-19 may be reluctant to respond to telephone calls from public health officials or to name their contacts, fearing stigma or quarantine, or they may be unwilling to opt into digital tracking due to privacy concerns [23-25]. A cornerstone of comprehensive contact tracing for infectious diseases is a community-based effort, including door-to-door visits to reach people who are unable or unwilling to engage via phone calls or digital platforms [26,27]. Face-to-face interactions between contact tracers with individuals with COVID-19 or their contacts may offer the opportunity to establish rapport and build trust needed to obtain personal information from reluctant individuals.

In June 2020, New York City (NYC) established the NYC COVID-19 Test & Trace Corps to develop and implement interventions to suppress COVID-19 transmission in NYC [28]. Beginning in June 2020, the contact tracing component of the Test & Trace Corps—Trace—attempted to reach people with COVID-19 and their contacts through telephone-based contact tracers. Between June and July 2020, Trace implemented 2 additional workflows with specialized staff to complement the efforts of the telephone-based tracers. These were efforts to (1) look for locating information of case-patients and contacts using Information Gatherers (IGs) when records lacked working telephone numbers; and (2) conduct home-based contact tracing using Community Engagement Specialists (CESs) when phone numbers were lacking or after unsuccessful telephone-based efforts. In this paper, we assess the contributions of the IGs and CESs to the NYC COVID-19 contact tracing efforts from July 2020-June 2021.

## Methods

### Study Population and Data Sources

**Figure 1:**
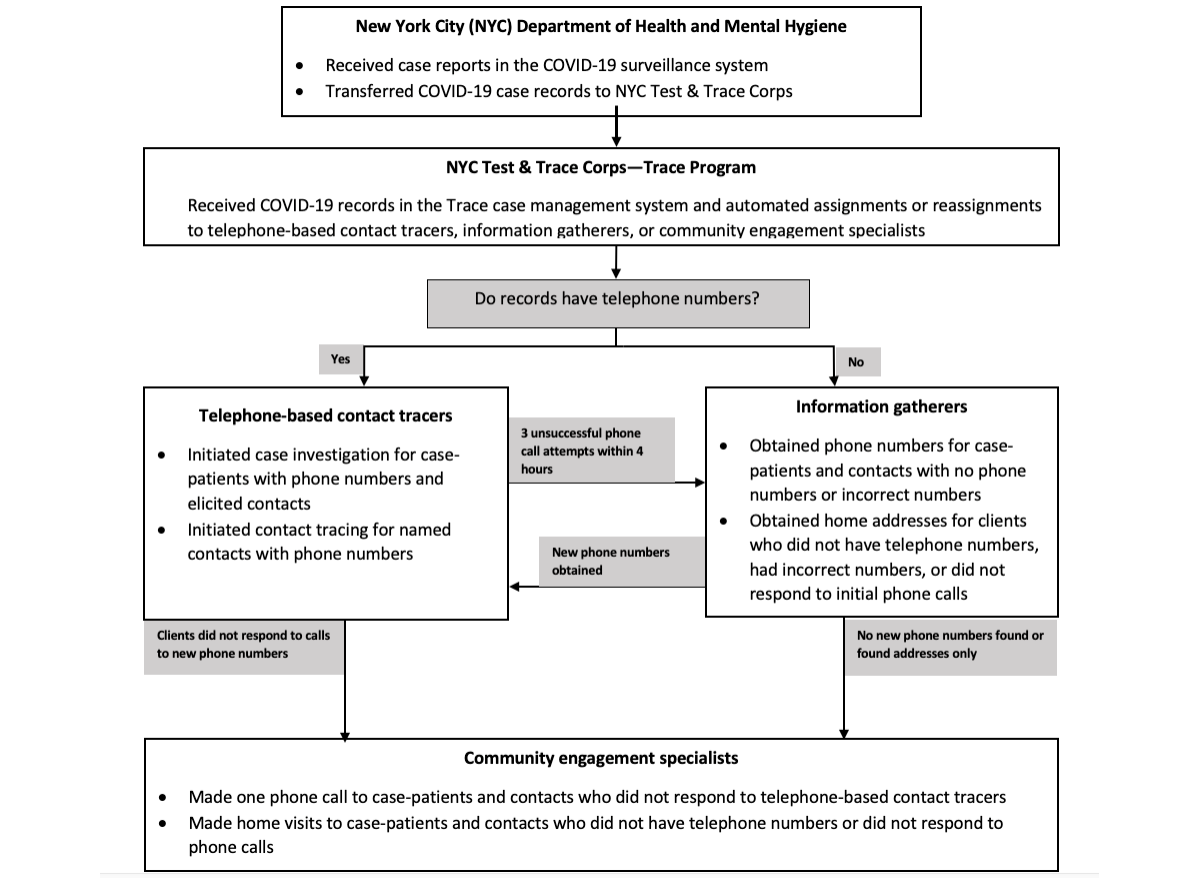
New York City Test & Trace Corps COVID-19 case investigation and contact tracing workflow.

All COVID-19 positive and negative results of tests performed by NYC laboratories and point-of-care testing sites were reported to the NYC Department of Health and Mental Hygiene’s (DOHMH’s) COVID-19 surveillance system. Daily, the DOHMH exported case records of confirmed or probable COVID-19 cases to the Trace case management system (Figure 1). To minimize the records with missing locating information, the DOHMH matched case records against available electronic medical record data systems of NYC medical institutions prior to data transfer to Trace. Data for our analysis were comprised of records forwarded to the Trace program from July 2020-June 2021. These records included the name and contact information of the ordering provider, demographic information of the case-patients (ie, name, phone number, address, and date of birth), date of specimen collection, and test type. We also analyzed records of the contacts named by case-patients. For each contact, contact tracers attempted to obtain name, phone number, address, date of birth, and the date of last exposure.

### Definitions

Case-patients were comprised of persons with probable or confirmed COVID-19 results or contacts with COVID-19 symptoms (or ‘symptomatic contacts’), even if the contacts had no reported COVID-19 test results [29]. Contacts were persons who came within 6 feet of people with COVID-19 during their infectious period for a cumulative total of ≥10 minutes over a 24-hour period [30]. The infectious period began 2 days before the onset of symptoms for case-patients or, if asymptomatic, 2 days before the specimen collection date of their COVID-19 positive test. We referred to case-patients or contacts as clients [31].

### Contact Tracing Workflow

Contact tracing encompassed case and contact investigations. Case investigation included the interview by a telephone-based tracer or CES to elicit contacts from case-patients, give isolation instructions, and make referrals for supportive services (eg, housing, groceries, and pet care). Contact investigation involved attempts to reach and interview the named contacts, inform them of their potential exposure to SARS-CoV-2, give recommendations for quarantine (ie, isolate, if symptomatic), and make referrals for supportive services.

Contact tracing workflow and the coordination of activities among telephone-based tracers, IGs, and CESs were managed within a software configured for Trace data management. Upon data transfer from the DOHMH, an automated algorithm assigned client records with telephone numbers to telephone-based tracers or to IGs if phone numbers were lacking (Figure 1). Furthermore, records were assigned to telephone-based tracers, IGs, or CESs based on the outcomes of the previous attempts. For example, if the telephone-based tracers could not reach clients at available phone numbers after 3 attempts within 4 hours of assignment, the records for those clients were then assigned to IGs to attempt to find new numbers. If IGs obtained new numbers, those records were reassigned to telephone-based tracers. If IGs obtained residential addresses only or clients did not respond to repeated outreach attempts by telephone-based tracers, those records were assigned to the CESs. Telephone-based tracers, IGs, and CESs recorded all interim and final outcomes they obtained in the Trace case management system in real time.

### Overview of IG Activities

For cases, IGs (n=74 at peak) called the reporting laboratories or ordering medical providers to obtain any available locating information (eg, telephone number and address) in their medical records. In addition, IGs manually searched CLEAR, a subscription service that collects public record information, including phone numbers and addresses, for locating information. For contacts, IGs did not contact the persons with COVID-19 who had named the contacts; rather, IGs used CLEAR to look for phone numbers or addresses. During the searches, IGs used clients’ first and last names, and full date of birth (ie, month, date, and 4-digit year) to confirm that information was being obtained for the referenced client. IGs did not perform provider or record searches if clients’ records were missing complete date of birth. During periods of high workloads relative to the number of IGs, IGs first prioritized information gathering for case-patients over contacts and same-day referrals over referrals from previous days.

IGs entered new phone numbers in the appropriate data fields in the Trace case management system, and the system queued the case or contact records for the telephone-based tracers. If only addresses were found, IGs updated the address field, and the records were then queued for the CESs. If neither phone numbers nor addresses were obtained, IGs made notes in the text field of the Trace case management system (July-November 2020) or assigned a final disposition of “unable to locate” new information (December 2020-June 2021).

### Overview of CES Activities

CESs’ COVID-19 prevention activities have been previously described [32]. In brief, CESs (n=540 at peak) performed in-person contact tracing and other COVID-19 prevention activities, such as the dissemination of COVID-19 information and sanitary supplies (eg, masks and hand sanitizers) at NYC schools, business establishments, and community settings. From July 2020-June 2021, the number of CESs assigned to perform contact tracing fluctuated daily (range: 192-492), depending on the need for them to engage in these other prioritized community-based COVID-19 prevention activities.

CESs’ contact tracing activities entailed making telephone calls and home visits to clients who did not have phone numbers or did not respond to telephone-based tracers. First, CES supervisors (n=50 at peak) manually assessed the records assigned to the CESs in the Trace case management system and made individual CES assignments, prioritizing case investigations over contact investigations. Supervisors also grouped clients by zip code, address, and telephone number to improve efficiency; for example, clients residing at the same address or with the same phone numbers were assigned to the same CES. At the beginning of their workday, CESs logged into the Trace case management system on their iPads and sequentially planned their outreach to clients. CESs first attempted phone calls to clients for whom telephone numbers were available, then made home visits to the addresses of clients who either did not respond to those phone calls or who had no telephone numbers on their record.

CESs received training on universal infection control practices and the proper use of personal protective equipment (eg, mask and face shield) and were instructed to conduct interviews outside clients’ front doors, standing at least 6 feet from the clients [32]. If clients were reached but could not complete phone calls or were located during a home visit but lacked privacy or space for physical distancing, CESs arranged for follow-up phone calls, encouraged clients to call Trace telephone-based tracers, or made another visit at a convenient time, within 24 hours. If clients’ addresses were confirmed but they were not found during home visits, CESs left letters asking them to call the Trace call line and if needed, arranged follow-up visits within 24 hours.

If CESs reached clients via phone calls or home visits, potential outcomes were as follows: (1) “completed interview,” (2) “declined to complete interview,” or (3) “unable to complete interview” (eg, assigned for call-back, unable to respond, currently outside NYC, or residing in congregate facility). If CESs did not reach clients via phone calls or home visits, the outcome was recorded as “unable to locate” (eg, wrong or nonexisting address, address not confirmed, or not home). The “unable to locate” disposition option was not available for CES use from July-November 2020.

### Data Analysis

We generated descriptive frequencies and proportions of the records referred to IGs and CESs from July 2020-June 2021 and summarized IG and CES workload and outcomes. Our analyses included only the records of clients who were referred to IGs or CESs for initial case and contact investigations. We deduplicated the records with multiple interviews and retained the first assignment and last outcome. We presented the proportions of select sociodemographic characteristics of clients by whether their records were ever referred to the IGs or CESs. Furthermore, we assessed the timeliness of the IG and CES activities by examining the median number of days and IQRs from the dates of referral (of cases) or identification (of contacts) to IGs or CESs to the date of initial attempt or final outcome (ie, interview or final disposition) and the date from initial attempt to final outcome. Data analysis was performed using R (version 3.5.2; R Foundation for Statistical Computing).

### Ethics Approval

Contact tracing data collection is part of routine public health surveillance and intervention and was determined to be nonresearch. Contact tracing, as a public health activity, was determined not to be research, in accordance with the federal human subject’s protection regulations at 45 Code of Federal Regulations 46.101c and 46.102d [33] and Centers for Disease Control and Prevention’s Guidelines for Defining Public Health Research and Public Health Non-Research (protection of human subjects, US Federal Code Title 45 Part 46) [34]. Participants voluntarily participated in the activities. Informed consent from participants was not required for contact tracing interview.

## Results

### Characteristics of Clients Referred to IGs or CESs

Case and contact demographics stratified by referral status to IGs and CESs are described in Table 1. Overall, 266,156 of 659,484 (40%) cases and 331,483 of 861,566 (38%) contacts were ever referred to the IGs and CESs over the period of July 2020-June 2021. Most of the referred case records (155,356/266,156, 59%) were from just 2 of the 5 NYC boroughs (ie, Brooklyn and Queens).

**Table 1:** Select characteristics of cases and contacts ever referred or not referred to information gatherers or community engagement specialists for case or contact investigation interview from July 2020-June 2021.

Characteristics		Cases (n=659,484)			Contacts (n=861,566)	
		Referred	Not referred	Referred		Not referred
Total, n (%)		266,156 (40.36)	393,328 (59.64)	331,483 (38.47)		530,083 (61.53)
**Borough, n (%)**						
	Bronx	45,793 (17.21)	69,025 (17.55)	35,536 (10.72)		77,882 (14.69)
	Brooklyn	81,809 (30.74)	111,167 (28.26)	49,464 (14.92)		118,907 (22.43)
	Manhattan	35,969 (13.51)	59,088 (15.02)	20,845 (6.29)		54,571 (10.29)
	Queens	73,547 (27.63)	111,091 (28.24)	51,207 (15.45)		121,420 (22.91)
	Staten Island	22,546 (8.47)	30,896 (7.86)	16,012 (4.83)		35,492 (6.70)
	Unknown	6492 (2.44)	12,061 (3.07)	158,419 (47.79)		121,811 (22.98)
**Race or ethnicity, n (%)**						
	Black (not Hispanic or Latino)	14,788 (5.56)	40,688 (10.34)	13,154 (3.97)		49,938 (9.42)
	White (not Hispanic or Latino)	23,823 (8.95)	61,369 (16)	17,319 (5.22)		69,809 (13.17)
	Hispanic or Latino	35,254 (13.25)	92,371 (23.48)	36,728 (11.08)		117,037 (22.08)
	Asian (not Hispanic or Latino)	11,847 (4.45)	28,285 (7.19)	9131 (2.75)		32,034 (6.04)
	Multiracial (not Hispanic or Latino)	775 (0.29)	2638 (0.67)	932 (0.28)		4174 (0.79)
	Native Hawaiian or Pacific Islander, Native American or Alaskan Native (not Hispanic or Latino)	262 (0.10)	675 (0.17)	249 (0.08)		766 (0.14)
	Did not identify with any race or ethnicity provided	2252 (0.85)	4926 (1.25)	2089 (0.63)		5725 (1.08)
	Unknown	177,155 (66.56)	162,376 (41.28)	251,881 (75.99)		250,600 (47.28)
Age (years), median (IQR), range		38 (24-56), 0-117	36 (25-52), 0-111	27 (13-45), 0-109		28 (12-46), 0-109
**Age group, n (%)**						
	0-12	24,621 (9.25)	35,504 (9.03)	48,197 (14.54)		120,006 (22.64)
	13-24	43,866 (16.48)	61,191 (15.55)	42,395 (12.79)		80,119 (15.11)
	25-44	90,463 (33.99)	153,299 (38.98)	57,748 (17.42)		130,253 (24.57)
	45-64	69,565 (26.14)	104,014 (26.45)	39,862 (12.03)		95,072 (17.93)
	≥65	37,180 (13.97)	39,255 (9.98)	12,874 (3.88)		27,023 (5.10)
	Unknown	461 (0.17)	65 (0.02)	130,407 (39.34)		77,610 (14.64)
**Gender identity, n (%)**						
	Male	126,417 (47.50)	171,135 (43.51)	40,590 (12.24)		119,129 (2247)
	Female	131,747 (49.50)	203,973 (51.86)	48,442 (14.61)		158,043 (29.81)
	Transgender, nonbinary, or queer	245 (0.09)	727 (0.18)	220 (0.07)		722 (0.14)
	Unknown	7747 (2.91)	17,493 (4.45)	242,231 (73.07)		252,189 (47.58)
**Preferred language, n (%)**						
	English	143,505 (53.92)	312,867 (79.54)	128,677 (38.82)		385,513 (72.73)
	Spanish	25,670 (9.64)	52,958 (13.46)	32,288 (9.74)		70,264 (13.26)
	Other	12,545 (4.71)	18,082 (4.60)	9048 (2.73)		15,510 (2.93)
	Unknown	84,436 (31.72)	9421 (2.40)	161,470 (48.71)		58,796 (11.09)
**Disability, n (%)**						
	Difficulty concentrating, remembering, or deciding	1260 (0.47)	4278 (1.09)	1516 (0.46)		5925 (1.12)
	Difficulty doing errands	372 (0.14)	951 (0.24)	333 (0.10)		983 (0.19)
	Difficulty dressing or bathing	112 (0.04)	266 (0.07)	100 (0.03)		270 (0.05)
	Difficulty hearing	922 (0.35)	1269 (0.32)	654 (0.20)		1226 (0.23)
	Difficulty seeing	2336 (0.88)	5008 (1.27)	2060 (0.62)		5589 (1.05)
	Difficulty walking or climbing stairs	1885 (0.71)	5577 (1.42)	137 (0.42)		4294 (0.81)
	Multiple disabilities	3916 (1.47)	9031 (2.30)	3037 (0.92)		8491 (1.60)
	No disability	80,968 (30.42)	199,514 (50.72)	70,946 (21.40)		223,498 (42.16)
	Unknown	174,385 (66.52)	167,434 (42.57)	251,458 (75.86)		279,807 (52.79)

### Workload and Outcomes of Referrals to IGs and CESs

**Figure 2:**
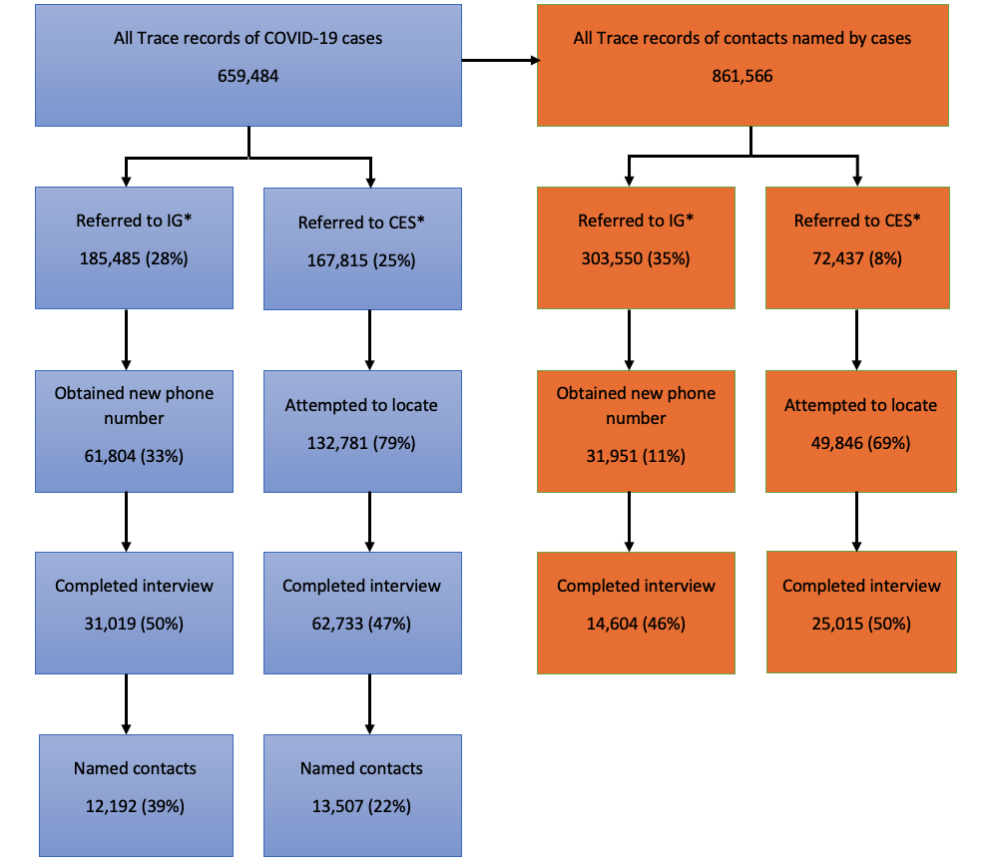
Workload and outcomes of referrals to information gatherers (IGs) and community engagement specialists (CESs), July 1, 2020-June 30, 2021. subsequent outcomes and proportions of subsequent steps were calculated based on the previous steps (eg, obtained new numbers were the proportions of records referred to IGs). The number of persons referred to IGs or CESs were not mutually exclusive. Some records may have been referred to both IGs and CESs work groups during the analysis period.

Figure 2 depicts the numbers and proportions of clients’ records referred to IGs and CESs from July 2020-June 2021 and the outcomes of those investigations.

Of the 659,484 Trace case records during this period, 185,485 (28%) were referred to IGs, and new phone numbers were obtained for 61,804 (33%) of the referred case-patient records. Subsequently, 31,019 (50%) of the case-patients with new phone numbers completed interviews, of whom 12,192 (39%) named contacts. During the same period, 303,550/861,566 (35%) contacts were referred to IGs. IGs obtained new phone numbers for 31,951 (11%) of the referred contact, of whom 14,604 (46%) completed interviews.

From July 2020-June 2021, 167,815/685,717 (24%) of Trace case records were referred to CESs. CESs attempted case investigation on 132,781 (79%) of the referrals; interviews were completed for 62,733 (47%) of the attempted referrals; and 13,507 (22%) of case-patients interviewed named contacts. Of the 861,566 contact records, 72,437 (8%) were referred to CESs. CESs investigated 49,846 (69%) of the referred contacts, and 25,015 (50%) of the contacts completed interviews.

**Figure 3:**
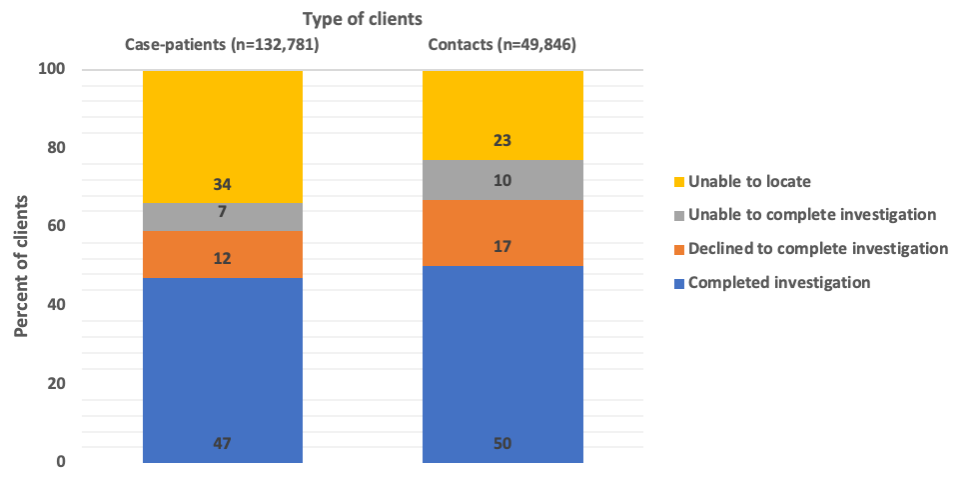
Outcomes of referrals attempted to locate by community engagement specialists for case and contact investigation, July 2020-June 2021. Number of cases: completed case investigation (62,733); declined to complete investigation (16,290); unable to complete investigation (9,310); unable to locate (44,448). Number of contacts: completed case investigation (25,015); declined to complete investigation (8,484); unable to complete investigation (5,104); unable to locate (11,243).

Among the 132,781 case investigations attempted by CESs, 44,448 (34%) of case-patients sought were never located through phone calls or home visits, 9,310 (7%) and 16,290 (12%) were located but were unable or declined to complete interviews, respectively (Figure 3). Among the 49,846 contact investigations attempted, CESs did not locate 11,243 (23%); 5,104 (10%) and 8,484 (17%) of persons located were unable or declined to complete interviews, respectively.

### Timeliness of IG and CES activities

**Figure 4:**
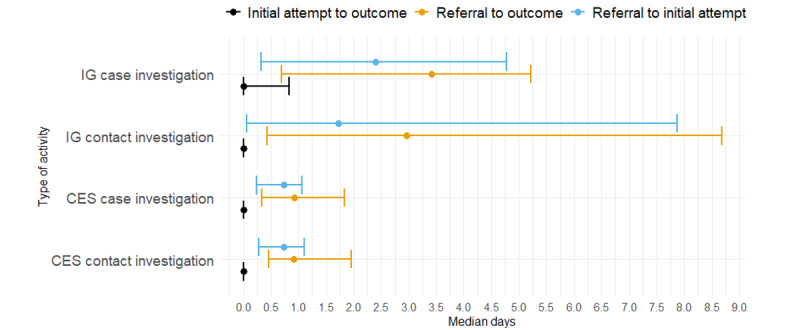
Timeliness measure of case and contact investigations referred and attempted by information gatherers (IGs) and community engagement specialists (CESs), July 2020-June 2021. Median days are from the dates of referral or initial attempts for case or contact investigations to outcomes (eg, found new number or interviewed clients). Error bars indicate IQRs of timeliness measures.

Among cases referred to IGs, the median interval was 2.4 (IQR 0.32-4.78) days from referral to the first attempt, 3.41 (IQR 0.7-5.22) days from referral to final outcome (eg, new phone number or declined to complete interviews), and 0 (IQR 0-0.83) days from first attempt to final outcome (Figure 4). Among contacts, the median interval was 1.72 (IQR 0.06-7.87) days from referral to IGs to first attempt, 2.96 (IQR 0.43-8.68) days from referral to final outcome, and 0 (IQR 0-0) days from first attempt to final outcome.

Among cases referred to CESs, the median interval was 0.74 (IQR 0.24-1.07) days from referral to first attempt to locate clients, 0.93 (IQR 0.33-1.83) days from referrals to final outcome, and 0 (IQR 0-0) days from first attempt to final outcome. Regarding contacts referred to CESs, the median intervals from referral to first attempt and to final outcome were 0.74 (IQR 0.28-1.11) days and 0.91 (IQR 0.46-1.96) days, respectively, and 0 (IQR 0-0) days from first attempts to final outcome.

## Discussion

We assessed the value added by information gathering and home visit workforces to manual telephone-based contact tracing. From July 2020-June 2021, despite the NYC DOHMH’s efforts to enrich the COVID-19 reports to the surveillance system with locating information from available electronic medical record sources, about 266,156/659,484 (40%) of case records and 331,483/861,566 (38%) contact records transferred to the Trace case management system lacked working telephone numbers or required home visit attempts to initiate contact tracing. This finding shows that missing locating information in reports from diagnostic providers and laboratories to public health disease surveillance systems delays or limits the already complex and multistepped manual contact tracing and supports the integration of digital proximity app-based contact tracing technique. Digital contact tracing using automated electronic information to identify individuals with new COVID-19 diagnosis and notify their exposed contacts has the potential to mitigate the lack of locating information on surveillance reports and shorten the time required for manual telephone contact notifications [8-10].

During the 1-year period of this study, the new phone numbers obtained by the IGs yielded interviews with an additional 31,019 case-patients and 14,604 contacts. The investigations attempted by the CESs added 62,733 completed interviews with case-patients and 25,015 with contacts. Furthermore, 12,192/31,019 (39%) and 13,507/63,733 (22%) of interviews with case-patients following the IG and CES interventions resulted in the identification of 12,192 and 13,507 contacts, respectively. Importantly, the median days for the completion of case and contact investigations was within 1 day of the IGs and CESs’ initial attempts to find new phone numbers or locate clients. Our results support the findings of a study of multiple US jurisdictions showing the important role of case investigation and contact tracing in reaching COVID-19 case-patients and contacts to implement COVID-19 prevention measures and curb ongoing disease transmission [6].

Information gathering [35] and face-to-face interactions [26,27,32] are core features of contact tracing for other infectious diseases, such as tuberculosis, HIV, and sexually transmitted infections. An effective contact tracing program aims to reach as many case-patients as possible to identify all potentially exposed contacts and then locate, evaluate, and educate those contacts on infection control. The unprecedented high volumes of COVID-19 incident cases required mass outreach and time-sensitive contact tracing strategies accomplished with telephone calls and digital platforms. However, the populations living in dense urban conditions, such as in NYC, are often most susceptible to SARS-Cov-2 acquisition [36,37], and among them are people with limited or unreliable access to telephone or digital communication services. Furthermore, mental and physical disabilities [38,39] or reluctance to share personal confidential information with strangers over phone calls could impede contact tracing on electronic platforms alone [40-42].

Our program used a 3-pronged approach, prioritizing phone calls when possible while simultaneously searching for locating information, or as a last recourse, making home visits. This strategy offers a contact tracing model that enhances the reach and yields of a contact tracing program and promotes equitable delivery of COVID-19 interventions [20-22]. We strived to minimize mistrust and communication gaps with our clients by recruiting CESs from NYC communities heavily impacted by COVID-19 and with language skills beyond English [32]. Our approaches can be adapted to jurisdictions’ resource levels and priorities. A jurisdiction could employ IGs alone to focus on obtaining missing locating information to increase case investigation and contact identification or use a small team of CESs to prioritize home visits for communities with the highest case counts or lowest response rates to telephone calls.

In addition to reaching the most people, another key factor to the success of contact tracing is the ability to reach people as quickly as possible following COVID-19 diagnosis or exposure [11,42]. Our results show that once our IGs and CESs initiated attempts to find new information or to locate clients, the median time to clients’ interviews was within 1 day. Therefore, the addition of the IG and CES workflows while increasing the breadth and yield of contact tracing outcomes did not markedly delay case investigations and contact notifications. For our program, this efficiency was enabled by the integrated Trace case management system, which allowed for real-time data sharing and automated algorithms for assignments and reassignments of investigations among the telephone-based tracers, IGs, and CESs.

Although the median times from referrals to CESs to their initial attempts or final outcomes were all within 1 day, we observed longer time intervals for the IGs (2->3 days) from referral to initial attempts or final outcomes. The reason for these delays were twofold. First CESs were required to complete all investigation within 24 hours. Second, from July-November 2020, the CESs and IGs lacked the ability to assign a final disposition code of “unable to locate” to clients, and these records remained on the IG queue for further investigation. Until the final disposition code was introduced, the IGs and CESs were instructed to sort and attempt assignments based on the most current date of referral.

Face-to-face interactions between contact tracers or health care practitioners with clients can help establish rapport and build trust, thus facilitating the sharing of confidential information [42-44] Although CESs reached the vast majority of the case-patients (88,333/132,781, 66%) and contacts (38,603/49,846, 77%) sought, fairly sizable proportions of each (25,600/132,781, 19%) and 13,588/49,846, 27%, respectively) either declined to be interviewed or postponed but never completed interviews. To address these refusals, our CESs’ standard operation procedures included the routine provision of brief COVID-19 prevention education materials and information on how to receive free services (eg, testing, vaccine when it became available, and social services) and instructions on safe isolation and quarantine.

About one-third of case-patients and one-fourth of contacts sought by CESs were never reached at their available telephone numbers or addresses. Prior reports on the outcomes of home-based contact tracing for COVID-19 are lacking. The rate of nonresponse among our study population highlights the importance of augmenting manual telephone contact tracing with digital contact tracing [8-10]; promoting mass testing and vaccination [45-47]; and widespread dissemination of COVID-19 prevention education through mass media campaigns, social network sites, and community settings [32,44,48-51]. In fact, during the study period, more than half of our CES workforce were regularly mobilized to participate in the dissemination of these COVID-19 prevention information and resources in community settings [32].

Our study is subject to several limitations. First, IGs and CESs could not attempt all the referrals due to the mounting caseload and with no increase in staffing. In particular, the number of CESs available for contact tracing was the lowest during the periods of COVID-19 resurgences in NYC when many CESs were reassigned to conduct community outreach to distribute COVID-19 sanitary supplies and COVID-19 information flyers to promote community COVID-19 testing sites. Second, despite the provision of an official contact tracing letter, some laboratory staff and medical providers did not give the IGs clients’ locating information, often citing the Health Insurance Portability and Accountability Act. This deficiency in public health case reporting requirement of full patient contact information and during IG follow-up impeded the completeness and timeliness of contact tracing. Third, there may have been some overlap between the number of interviews or additional contacts identified among the IG and CES outcomes. Some clients with new telephone numbers may have been forwarded to CESs for home visits. Fourth, missing data on clients’ sociodemographic characteristics prevented us from assessing the potential differences between the clients who were reached by telephone-based tracers and those referred to the IGs or CESs.

Manual telephone contact tracing even when augmented with information gathering and home visits faces limitations, including being labor and time-intensive and insufficient staffing. Although digital contact tracing has the potential to rapidly notify exposed contacts and provide risk reduction information and resources, it relies on mass ownership and adoption of the digital platforms and minimal concerns of individuals for their privacy. These limitations underscore the importance of generalized COVID-19 prevention measures, such as universal self-masking, sanitary supplies, vaccination, and antiviral treatment for severe illness.

Our program’s approaches demonstrate that the efforts of manual telephone-based tracers can be complemented by information gathering and in-person contact tracing to achieve increases in the number of people reached for case investigation and contact identification, and therefore, in contact notification. Missing or incomplete telephone numbers and locating information on surveillance reports initially sent to the NYC DOHMH from diagnostic providers and laboratories show the need for improvements in data collections at the time of diagnosis or the completeness of data reported by providers to health departments. In settings with limited resources for information gathering and home visits, targeted applications of these strategies could focus on geographic areas or demographics with the highest incidence of COVID-19 or low contact tracing participation rates.

## Abbreviations

CES: community engagement specialist
DOHMH: Department of Health and Mental Hygiene
IG: information gatherer
NYC: New York City

## Data Availability

The data sets generated or analyzed during this study are available from the corresponding author on reasonable request.
